# Comparison of SHF and SSF of wet exploded corn stover and loblolly pine using in-house enzymes produced from *T. reesei* RUT C30 and *A. saccharolyticus*

**DOI:** 10.1186/2193-1801-3-516

**Published:** 2014-09-11

**Authors:** Vandana Rana, Anahita D Eckard, Birgitte K Ahring

**Affiliations:** Bioproducts, Sciences and Engineering Laboratory (BSEL), Washington State University, 2710 Crimson Way, Richland, WA 99354-1671 USA

**Keywords:** Wet explosion, Wet exploded corn stover, Wet exploded loblolly pine, T. reesei Rut C30, A. saccharolyticus, Fermentation, SHF, SSF

## Abstract

The aim of the present study was to compare bioethanol production from wet exploded corn stover (WECS) and loblolly pine (WELP) hydrolyzed with in-house and commercial enzymes and fermented separately (SHF) and simultaneously (SSF). In-house enzymes produced from *Trichoderma reesei*, RUT-C30 and a novel fungal strain, *Aspergillus saccharolyticus* were loaded as 5 and 15 FPU/g glucan and supplemented with 10 and 30 CBU/g glucan, respectively. For hydrolysis and fermentation, slurries of WECS and WELP at 5 and 10% (w/w) solids loading (SL) were utilized. *Saccharomyces cerevisae* was used for ethanol fermentation at 33°C. Maximally, 15.6 g/L and 13.4 g/L (corresponding to theoretical ethanol yield of 76% and 67%, respectively) were achieved in SSF process from WECS and WELP, respectively at 5% SL and 15 FPU/g glucan loading of in-house enzymes. Ethanol concentrations in all cases were higher for SSF compared to SHF under same conditions. A cross comparison of SSF with commercial enzymes (Celluclast 1.5 L + Novozym 188) showed highest ethanol concentration of 17.3 g/L and 15.4 g/L (corresponding to theoretical ethanol yield of 84% and 77%, respectively) from WECS and WELP, respectively at 5% SL and 15 FPU/g glucan. These findings demonstrated that in-house enzymes were comparable to commercial enzymes as these fungi produced other lignocellulolytic enzymes beyond cellulase and hence enhanced the overall enzyme activity.

## Introduction

The cost and hydrolytic efficiency of lignocellulolytic enzymes are critical parameters in bioethanol production (Himmel et al. 1999; Lynd et al. 2005; Galbe and Zacchi 2002; Kovács et al. 2009). Thus, it is necessary to find microorganisms with better cellulolytic properties. The filamentous fungus *Trichoderma reesei* RUT-C30 has already been established as a producer of cellulases and hemicellulases which are extensively used in paper, pulp, food, feed and textile industries and recently, *Trichoderma reesei* RUT-C30 has been explored for its lignocellulolytic properties and hence is used in saccharification of lignocellulosic biomass to monomeric sugars for production biofuels (Bouws et al. 2008; Kumar et al. 2008).

Ethanol production from lignocellulosic biomass involves three core steps: i) Pretreatment ii) Enzymatic hydrolysis or saccharification iii) Fermentation. Hydrolysis of sugars followed by fermentation step is called separate hydrolysis and fermentation (SHF). As an alternative these hydrolysis and fermentation steps can be merged together in one process known as simultaneous saccharification and fermentation (SSF). There are pros and cons associated with both of these processes. An advantage of SHF is that enzymes and yeast can each operate at their optimal conditions, e.g. with respect to temperature, However, SHF has the disadvantage that inhibitory hydrolysis products accumulate, decreasing reaction rates (Stenberg et al. 2000; Xiao et al. 2004). In SSF, temperature is not optimal for cellulases and, therefore, the rate of hydrolysis is slow, but hydrolysis products can be consumed as they are formed due to fermentation, thus avoiding the inhibition seen with SHF (Ballesteros et al. 2004; Olsson et al. 2006). Furthermore, ethanol in the fermentation broth prevents significant microbial contamination. Another advantage of SSF is that the process integration of hydrolysis and fermentation in one reactor reduces the overall capital cost.

Although these processes to produce bioethanol are promising, the cost of added enzymes is substantial in many designs (Dutta et al. 2010; Kazi et al. 2010). One approach to reducing costs is use of at-site produced crude enzymes, which avoid costs for purification and transport (Schell et al. 1990; McMillan 1997). Another approach to achieving cost-savings is to eliminate filtration and washing after pretreatment, resulting in lower capital costs, less dilution, and higher product concentrations. However, pretreated slurry contains some sugar and lignin degradation products which are inhibitory to enzymes and yeast leading to decreased fermentation rates. Thus, it is important to employ a robust fermenting microorganism such as *Saccharomyces cerevisiae*, which shows high ethanol productivity and high tolerance to these inhibitory compounds (Olsson and Hahn-Hägerdal 1993). In a previous study, it was found that some of these fermentation inhibitors can be completely metabolized by the yeast during SSF by metabolic redox reactions (Tomás-Pejó et al. 2008). In addition to the problems due to mass transfer in viscous pretreated material (Ingesson et al. 2001; Wen et al. 2004; Klinke et al. 2004), these inhibitory compounds restrict the maximum allowable water insoluble solid (WIS) in SSF and SHF processes (Linde et al. 2007).

In this study, SHF and SSF were performed on wet exploded corn stover and loblolly pine using in-house produced enzymes at two different concentrations of WIS and at two different enzyme loadings to determine which process is the most suited for the ethanol production. The influence of solid and enzyme loadings on ethanol yield were further evaluated using both in-house and commercial enzymes.

This research work confirms the novelty as no study has been reported up until now on the integration of process of in-house cellulase production by *Trichoderma reesei* Rut C30 & *Aspergillus saccharolyticus* and ethanol production using those in-house produced cellulase cocktail from wet explosion pretreated corn stover and loblolly pine.

## Materials and methods

### Raw material

Quarter inch corn stover and loblolly pine were kindly obtained from Iowa State University. Raw materials were milled to 2 mm size for compositional analysis and pretreatment. Composition of raw corn stover (% dry matter basis) was as follows: glucan 38.7%, xylan 25.2%, galactan 1.83%, arabinan 2.85%, mannan 0.38%, lignin 17.5%, ash 2.6% and composition of raw loblolly was; glucan 35.9%, xylan 8.5%, galactan 2.5%, arabinan 1.6%, mannan 8.2%, lignin 30.7%, ash 0.8%.

### Wet explosion pretreatment

Wet explosion pretreatment was performed in a wet explosion pretreatment unit with a 10 L reactor described previously (Rana et al. 2012). In brief the corn stover was subject to pretreatment at 170°C for 20 min with 79.8 psi oxygen and loblolly pine was pretreated at 175°C for 24 min at 79.8 psi oxygen. These conditions were selected according to previous studies (data not shown) and based on optimal process conditions and sugar yields after enzymatic hydrolysis (Rana et al. 2013). Whole pretreated slurries were stored at 4°C for further studies.

A portion of pretreated slurry was divided into two fractions: (i) solid fraction or water insoluble solids (WIS) and (ii) liquid fraction or prehydrolyzate. To obtain the WIS, the solid fraction was washed with water multiple times and dried at 30°C for 4 days to obtain moisture content less than 10%. Both fractions were analyzed for sugars, lignin and degradation products.

### Microorganisms

Mutant fungi, *Trichoderma reesei* Rut-C30, and a novel fungi *Aspergillus saccharolyticus* (CBS 127449) were used for cellulase and β-glucosidase production, respectively as previously described (Rana et al. 2014).

### Preparation of biomass for enzyme production

Corn stover was used as a substrate for cellulase production from *T. reesei* Rut-C30 and *A. saccharolyticus*. Quarter inch corn stover was milled to 1.5 mm and pretreated with wet explosion and subsequent subjected to alkali for *T. reesei* and just wet exploded for *A. saccharolyticus*. Pretreatment was conducted at the 100 L pilot plant facility of Washington State University using 25% (D.M.) solid loading at 175°C, for 25 min and an oxygen flow of 6.0%. A portion of the wet exploded slurry was separated and liquor was used for *A. saccharolyticus*. The remaining portion of the pretreated biomass (wet exploded) was used for alkali pretreatment by subsequent supplementation with 1% w/v NaOH at a solid loading of 10% (w/v). Temperature was kept between 95–100°C for 5 h and pH of the resulting slurry was ~11.0. Combined wet explosion-alkali pretreated slurry was fractionated to give a liquor and pretreated biomass after centrifugation and subsequently dehydrated with a screw press before being used for enzyme production. Compositional analysis of pretreated biomass was conducted according to the NREL/TP-510-42618 procedure.

### Preparation of inoculum

Inoculum cultures for fungal strain *T. reesei* RUT-C30 were prepared from −80°C glycerol stocks on agar plates containing 39 g L^−1^ yeast extract potato dextrose (YPD) and incubated statically at 30°C for 120 h. Colonies with surfaces were picked and went through consecutive transfers and streaking on the medium to obtain pure colonies. The composition of the medium was 30 g/l wheat bran, 25 g/l corn steep liquor (CSL), 5 g/l Avicel, 50 g/l glucose, 30 g/l peptone and 5 g/l yeast extract in a flask with a total volume of 150 ml. 50 ml of autoclaved mineral solution was added to the media with the composition: 0.3 g/l of MgSO_4_.7H_2_O, 4 g/l of KH_2_PO_4_, 2 g/l (NH_4_)_2_SO_4_ and 0.3 g/l of CaCl_2_.2H_2_O. Media was inoculated with 3 ml of spore’s suspension, cooled, and the cultures were grown for 48 h at 30°C in a rotary shake at 140 RPM.

Inoculation media for *A. saccharolyticus* was prepared in 1000 ml shake flasks with the active volume of 200 ml. The composition of inoculation media was 40 g/l wheat bran, 40 g/l corn steep liquor (CSL), 4 g/l peptone, 2 g/l yeast extract, 2 g/l casamino acids, 12 g/l NaNO_3_, 1 g/l KCl, 1 g/l MgSO_4_ 7H_2_O, 3 g/l KH_2_PO_4_, 0.1 g/l Na_4_ EDTA, 4.5 mg/l ZnSO_4_ 7H_2_O, 22 mg/l H_3_BO_3_, 10 mg/l MnCl_2_ 4H_2_O, 10 mg/l FeSO_4_ 7H_2_O, 3.4 mg CoCl_2_ 6H_2_O, 3.2 mg/l CuSO_4_ 5H_2_O, 0.17 mg/l Na_2_MoO_4_ 2H_2_O. The media was sterilized by autoclave at 121°C, for 15 min. The inoculation media was left to equilibrate in a shake incubator set to operate at 28°C, 140 RPM. After equilibration the media was inoculated using 3 ml spore suspension and the cultures were grown for 48 h at 30°C and pH 4.8 in a rotary shaker.

### Enzyme production

For enzyme production from *T. reesei* Rut-C30, 150 mL of the pre-culture was used to inoculate a 3 L stirred reactors. The airflow was kept at 1.2 L/min and the stirrer speed at 800 rpm. The culture conditions were maintained at 28°C and at pH 3.75 by automatic addition of 5 NH_4_OH. The medium composition was as follows: 2.5% (DM) wet exploded-alkali pretreated corn stover, 2.5% corn steep liquor, 2.5% wheat bran, 0.05% yeast extract, 0.3% peptone, 5% corn mash (liquefied with α-amylase to contain dextrin). The final volume was 1.8 L and the cells were cultured for 24 h after which the corn mash (with 33% M.C.) was fed into the reactor in a fed-batch mode with an average initial dilution rate of 0.007 h^-1^. For saccharification of maltodextrin to D-glucose in corn mash, 300 μl of gluco-amylase per liter of mash was mixed in the feed. To prevent bacterial growth during fermentation, 0.1% v/v of kanamycin was added to both the reactor and the feed bottle.

β-glucosidase production from *A. saccharolyticus* was conducted in a stirred reactor (5 L) equipped with online control system for adjustment of pH, temperature, antifoam, agitation and dissolved oxygen level (DO). The fermentation was performed in a feed batch setup. 800 ml of inoculation media was used as a startup media, the media was added to the 5 L reactor for sterilization by autoclave at 121°C for 15 min. After sterilization the reactor was stabilized for 4 hours at 28°C, pH 4.8, 800 RPM, aeration set-point was 0.7 L air/L/m. The reactor was seeded using 200 ml of inoculum as seed culture and, at 24 hours of operation the feed was started at 22 ml/h, total fermentation time was 8 days. The enzyme containing liquid was filtered and consequently concentrated 10 times by rotary vacuum evaporation.

### Enzymes and activities

Celluclast 1.5 L and Novozyme 188, were obtained from Sigma Aldrich. Filter-paper and Carboxymethyl cellulose activities were used as a measure of cellulase activity. FPA 4.49 FPU/mL and CMCase to 20.6 U/mL was measured after 7 days of fermentation. β-glucosidase activity on pNPG was measured as 4.77 U/mL and xylanase activity as 6.61 nkat/mL. Commercial cellulase (celluclast 1.5 L) showed 81.8 FPU/mL filter paper activity, β-glucosidase activity of 58.66 U/mL and xylanase activity 107.3 nkat/mL. PNPG activities (β-glucosidase) in *A. saccharolyticus* and in commercial enzyme Novozym 188 were 339.9 U/mL and 698.3 U/mL, respectively.

### Yeast cultivation

*S. cerevisiae*, a commercially available Baker’s yeast was used for fermentation. Nutrient media contained glucose, 20 g/L; yeast extract, 10 g/L; proteose peptone, 20 g/L and (NH_4_)_2_SO_4_, 3.42 g/L were autoclaved at 121°C for 20 min, cooled to room temperature and dry yeast cells were added to the nutrient medium. Four shake flasks with 100 mL working volume were incubated for 24 h at 33°C in a rotary shaker at 150 rpm.

### Separate hydrolysis and fermentation (SHF) with in-house enzymes

Enzymatic saccharification was performed at 5 and 10% solids loading for 72 hours prior to the fermentation. Enzymes produced in-house from *T. reesei* and *A. saccharolyticus* mixture were added in two different loadings (i) 5 FPU/g glucan + 10 CBU/g glucan and (ii) 15 FPU/g glucan + 30 CBU/g glucan, respectively. Sterile salt solution [(NH_4_)_2_HPO_4_, 1.3 g/L; MgSO_4_.7H_2_O, 0.01 g/L; KH_2_PO_4_, 0.6 g/L, 1% antibiotic kanamycin] needed for the fermentation were added during hydrolysis step to maintain the desired solid loading (5 and 10%) throughout the process and also avoid further dilution during fermentation due to addition of salt solution. All hydrolysis runs were carried out in 250 mL shake flasks at 50°C, pH 5 and 150 rpm for 72 hours.

The fermentation was started by addition of 1 mL of cell suspension of yeast yielding a cell concentration of 2 g L^−1^ dry weight (DW). The flasks were placed in a rotary shaker and operated at a shaker speed of 150 rpm at a temperature of 33°C for 96 hrs. 1.5 ml samples were withdrawn periodically for analysis using a sterile syringe attached to a sampling tube.

### Simultaneous saccharification and fermentation (SSF) with in-house enzymes

SSF experiments were conducted at similar experimental conditions as SHF. Pretreated slurry (5% and 10% WIS of WECS and WELP), sterile salt solution (5 mL [(NH_4_)_2_HPO_4_, 1.3 g/L; MgSO_4_.7H_2_O, 0.01 g/L; KH_2_PO_4_, 0.6 g/L, 1% antibiotic kanamycin], enzymes (5 and 15 FPU/g glucan) and yeast cell suspension (cell concentration of 2 gL^-1^ DW) were added in 250 mL shake flasks at a final working volume of 150 mL and kept at 33°C for 96 h.

Periodically, 1.5 ml samples were taken and centrifuged and clear samples were analyzed for ethanol, residual sugars and glycerol using HPLC. Dry mass of the yeast inoculum was determined according to NREL/TP-510-42630 protocol. The volumetric ethanol productivity was calculated based on ethanol concentration after 96 hours divided by total hours. Theoretical ethanol yield was calculated based on Eq. (1)
1

### Analytical methods

The pretreated materials were analyzed using NREL’s laboratory analytical procedure for determination of structural carbohydrates and lignin in biomass (NREL/TP-510-42618). Dry matter content of the slurry was determined by drying the material overnight at 105°C. Sugars, sugars degradation products (furfural, 5-hydroxymethylfurfural (HMF) and acetic acid) and ethanol were measured using high performance liquid chromatography, HPLC (Agilent Technologies, Santa Clara, CA) using an Aminex ion exclusion HPX-87H cation-exchange column (Bio-Rad, Hercules, CA) at 60°C with a flow rate of 0.6 ml/min with 5 mM sulfuric acid as the mobile phase. Peaks were identified using refractive index (RI) detector at 60°C.

Dry cell mass of the yeast inoculum was measured according to NREL/TP-510-42630 by centrifuging 8 ml of inoculum at 4000 rpm for 5 min. Supernatant was discarded and pellets were washed twice with 5 ml deionized water and centrifuged. After washing the pellets were dried in a convection oven at 105°C until constant weight was obtained.

## Results

The pretreated corn stover and loblolly pine were saccharified using in-house produced cellulases and commercial cellulases and were used for fermentation by *Saccharomyces cerevisae*. Fermentation yields using in-house and commercial enzymes were compared.

### Fermentation substrate-wet exploded corn stover & loblolly pine

**Figure 1:**
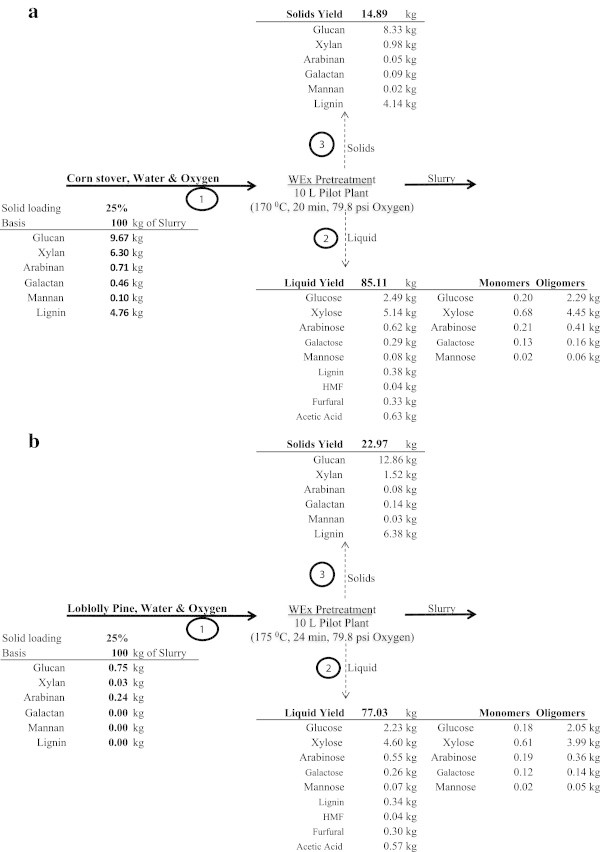
**Material balance of wet explosion pretreatment. (a)** Corn Stover. **(b)** Loblolly Pine.

Wet explosion is a combination of wet oxidation and steam explosion in which wet oxidized biomass is suddenly depressurized/exploded (Rana et al. 2012). Slurries with total solids contents of 23% and 25% (w/w) were obtained after wet explosion pretreatment of corn stover and loblolly pine, respectively. The mass balance of the pretreated materials is given in Figure 1a & b. 2.49 kg of glucose corresponding to 23% overall conversion was obtained from wet exploded corn stover and, 2.23 kg of glucose corresponding to 21% conversion from wet exploded loblolly pine. Resulting sugars as well as sugar degradation products are also shown in Figure 1. It can be concluded that most of hemicellulosic sugars were solubilized and are present mainly in oligomeric form. Very little lignin was solubilized (1% solubilized) during the wet explosion pretreatment. Furfural 3.99 g/L and 1.29 g/L from CS and LP respectively was formed due to pentose degradation and HMF 0.5 g/L and 0.71 g/L from hexose degradation in pretreated liquor of corn stover and loblolly pine, respectively. Acetic acid 7.57 g/L and 3.58 g/L were released from corn stover and loblolly pine, respectively due to solubilization of acetyl groups present in the hemicellulose. These degradation compounds are considered inhibitory for the fermentation. These inhibitors have been previously been identified in corn stover, wheat straw, barley straw (García-Aparicio et al. 2006; Öhgren et al. 2005; Oliva et al. 2003).

### SHF of WECS and WELP with enzyme mixtures produced in-house

**Figure 2:**
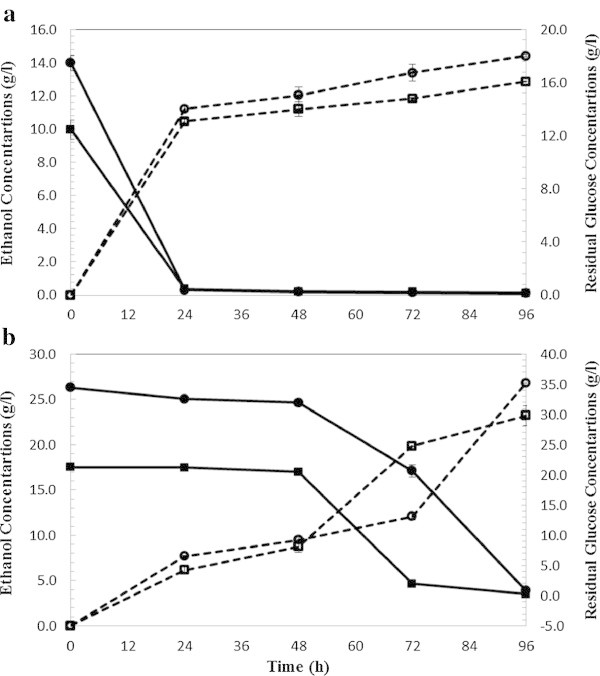
**SSF ethanol production (dashed lines) and residual glucose (bold lines) from wet exploded Loblolly pine. (a)** At 5% solid loading and enzyme loading 5 FPU (squared label) and 15 FPU/g glucan (circled label). **(b)** At 10% solid loading and enzyme loading 5 FPU (squared label) and 15 FPU/g glucan (circled label).

**Figure 3:**
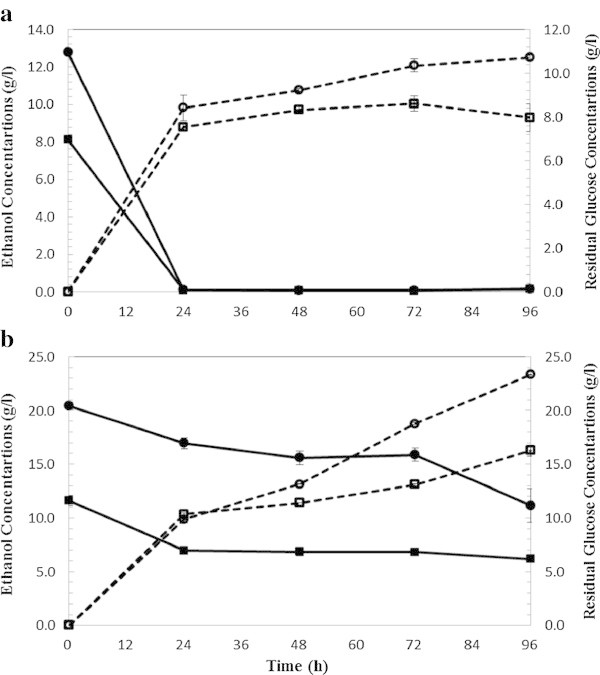
**SHF ethanol production (dashed lines) and residual glucose (bold lines) from wet exploded Loblolly pine. (a) At** 5% solid loading and enzyme loading 5 FPU (squared label) and 15 FPU/g glucan (circled label). **(b) At** 10% solid loading and enzyme loading 5 FPU (squared label) and 15 FPU/g glucan (circled label).

SHF with two different solids loadings (5% and 10%) at two enzymes loadings (5 and 15 FPU/g glucan), was evaluated with regard to ethanol yield for WECS and WELP using in-house enzymes produced and commercial enzymes as shown in Tables 1 and 2 respectively. Figures 2 and 3 show the final glucose and ethanol concentrations and glucose consumption rate over the period of 96 hours of fermentation of wet exploded corn stover.

**Table 1:** **Fermentation using in-house enzymes produced from** ***T. reesei*** **and** ***A. saccharolyticus***

	Enzyme loading (FPU/g glucan)	Solid loading (g/L)	Ethanol conc. (g/L)	Glycerol conc. (g/l)	***Y***_***EtOH/Glu***_(%)	Qp _EtOH/Gluc_ (g/l.h)
**Corn stover - (SHF)**	5	50	12.86 ± 0.02	0.30 ± 0.03	62.61	0.13
	15	50	14.41 ± 0.08	0.69 ± 0.05	70.19	0.15
	5	100	23.23 ± 1.10	1.28 ± 0.25	56.56	0.24
	15	100	26.83 ± 0.00	1.40 ± 0.00	65.33	0.28
**Corn stover - (SSF)**	5	50	13.59 ± 0.08	0.35 ± 0.01	66.19	0.14
	15	50	15.60 ± 0.12	0.56 ± 0.01	75.98	0.16
	5	100	25.68 ± 0.19	1.02 ± 0.01	62.53	0.27
	15	100	28.42 ± 1.09	1.35 ± 0.06	69.21	0.30
**Loblolly pine - (SHF)**	5	50	9.29 ± 0.74	0.27 ± 0.05	46.51	0.10
	15	50	12.50 ± 0.13	0.27 ± 0.00	62.54	0.13
	5	100	16.29 ± 0.51	0.53 ± 0.00	40.77	0.17
	15	100	23.33 ± 0.01	0.56 ± 0.03	58.37	0.24
**Lobllolly pine - (SSF)**	5	50	11.93 ± 0.01	0.33 ± 0.00	59.70	0.12
	15	50	13.40 ± 0.12	0.40 ± 0.01	67.07	0.14
	5	100	19.96 ± 0.34	0.48 ± 0.01	49.96	0.21
	15	100	24.98 ± 0.32	0.56 ± 0.03	62.51	0.26

**Table 2:** **Fermentation using commercial enzymes (Celluclast 1.5 L and Novozym 188)**

	Enzyme loading (FPU/g glucan)	Solid loading (%)	Ethanol concentration (g/L)	Glycerol (g/l)	***Y***_***EtOH/Glu***_(%)	Qp _EtOH/Gluc_ (g/l.h)
**Corn stover - (SSF)**	5	5	13.83 ± 0.49	0.99 ± 0.34	67.37	0.14
	15	5	17.28 ± 1.31	0.77 ± 0.00	84.14	0.18
	5	10	27.51 ± 1.49	1.33 ± 0.01	66.98	0.29
	15	10	32.53 ± 0.15	0.36 ± 0.01	79.21	0.34
**Loblolly pine - (SSF)**	5	5	12.40 ± 0.04	0.04 ± 0.00	62.04	0.13
	15	5	15.42 ± 0.01	0.04 ± 0.00	77.19	0.16
	5	10	20.98 ± 0.21	0.15 ± 0.06	52.50	0.22
	15	10	27.08 ± 0.08	0.12 ± 0.01	67.77	0.28

For SHF of WECS, at 5% solid loading (Figure 2a), the maximum ethanol concentration was 12.86 and 14.41 g/L in CS-1 and CS-2, respectively. All the glucose was exhausted during the first 24 hours and was consumed at a linear rate, slightly decreasing with time. After 96 h a residual glucose concentration of 1 g/L was found in CS-1 and CS-2 hydrolyzates. Glycerol is one of the major by-products during biomass (glucose) conversion to ethanol (Zaldivar et al. 2005) and at the stationary phase (no cell growth) glycerol production ceases. 0.3 g/L and 0.7 g/L of glycerol were formed respectively in CS-1 and CS-2 demonstrating that glucose was mainly consumed for the ethanol production.Increased solids loading and decreased enzyme loading (10%, 5 FPU/g glucan) lowered the ethanol yield in SHF of WECS. At 10% solids loading and cellulase loading of 5 FPU/g glucan (CS-3) (Figure 2b), an ethanol concentration of 23.23 g/L corresponding to the ethanol yield of 56.6% was achieved. With increasing enzyme loading to 15 FPU/g glucan (CS-4), resulted in an ethanol concentration of 26.83 g/L corresponding to a yield of 65.3%. Glucose was exhausted gradually; only 0.24 and 0.82 g/L of glucose remained in CS-3 and CS-4 after 96 hours of fermentation. Glycerol concentrations of 1.3 g/L and 1.4 g/L resulted, respectively, in CS-3 and CS-4.Figure 3 shows the ethanol production and glucose consumption during 96 hours of SHF of loblolly pine at 5% SL (Figure 3a) and 10% SL (Figure 3b). At 5% solid loading (Figure 3a), the maximum ethanol concentrations were 9.29 and 11.03 g/L in LP-1 (5 FPU/g glucan) and LP-2 (15 FPU/g glucan), respectively after 96 hours. All the glucose was exhausted during the first 24 hours and was consumed at a linear rate. Less than 0.5 g/L of glucose remained in LP-1 and LP-2 hydrolyzate at the completion of the fermentation. 0.26 g/L and 0.27 g/L of glycerol were formed, respectively, in LP-1 and LP-2 demonstrating that glucose was mainly consumed for ethanol production. At 10% solids loading (Figure 3b), the maximum ethanol concentrations were 16.29 and 23.33 g/L in LP-3 (5FPU/g glucan) and LP-4 (15 FPU/g glucan), respectively. Glucose consumption was slower compared to LP-1 and LP-2 and 6.19 and 11.15 g/L of glucose remained in LP-3 and LP-4 after 96 hours of fermentation. 0.53 g/L and 0.56 g/L of glycerol were formed respectively in LP-3 and LP-4.

### SSF of WECS and WELP with enzyme mixtures produced on-site

**Figure 4:**
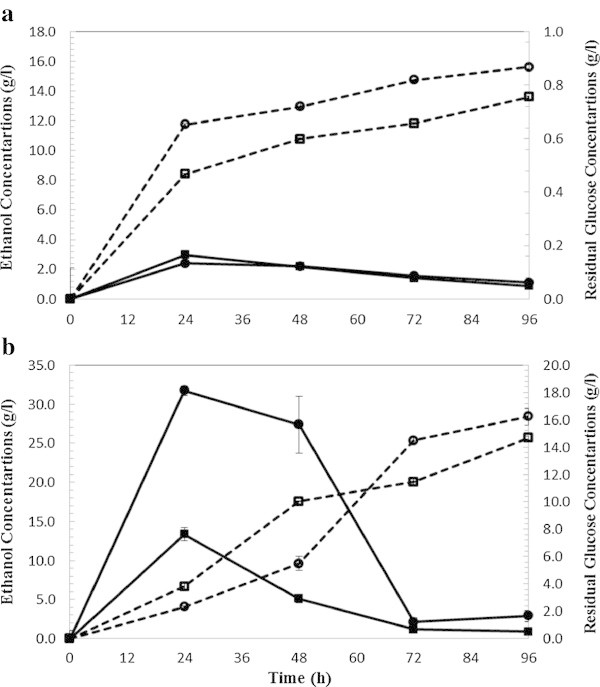
**SSF ethanol production (dashed lines) and residual glucose (bold lines) from wet exploded corn stover. (a)** At 5% solid loading and enzyme loading 5 FPU (squared label) and 15 FPU/g glucan (circled label). **(b)** At 10% solid loading and enzyme loading 5 FPU (squared label) and 15 FPU/g glucan (circled label).

**Figure 5:**
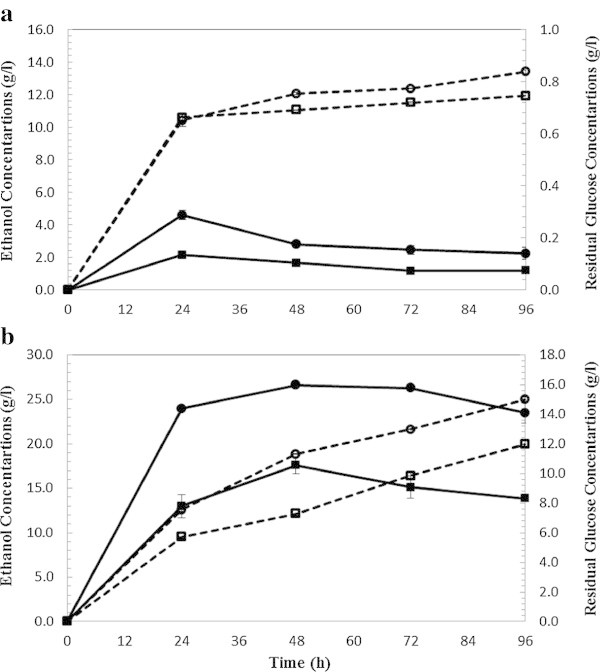
**SSF ethanol production (dashed lines) and residual glucose (bold lines) from wet exploded corn stover using commercial enzymes. (a)** At 5% solid loading and enzyme loading 5 FPU (squared label) and 15 FPU/g glucan (circled label**). (b)** At 10% solid loading and enzyme loading 5 FPU (squared label) and 15 FPU/g glucan (circled label).

SSF experiments were conducted with similar process condition, 5% and 10% solid loadings and 5 and 15 FPU/g glucan enzyme loadings of on-site produced enzymes for fermentation of WECS and WELP. Figure 4 shows ethanol production and glucose consumption over the period of 96 hours of fermentation of WECS. At 5% solids loading (Figure 4a), the maximum ethanol concentration was 13.59 and 15.60 g/L in CS-1 and CS-2, respectively. Less than 1 g/L of glucose remained in CS-1 and CS-2 after fermentation. 0.35 g/L and 0.56 g/L of glycerol were formed in CS-1 and CS-2, respectively at the end of the fermentation. The maximum ethanol concentration was 25.68 and 28.42 g/L in CS-3 and CS-4, respectively. Glucose was exhausted gradually; only 0.50 and 1.67 g/L of glucose remained in CS-3 and CS-4 after 96 hours of fermentation. 1.01 g/L and 1.35 g/L of glycerol were formed respectively in CS-3 and CS-4.Figure 5 shows ethanol production and glucose consumption over the period of 96 hours of fermentation of loblolly pine. At 5% solids loading (Figure 5a), the maximum ethanol concentration was 11.93 and 13.40 g/L in LP-1 and LP-2, respectively. Less than 0.5 g/L of glucose remained in LP-1 and LP-2 after fermentation. 0.33 g/L and 0.40 g/L of glycerol were formed respectively in LP-1 and LP-2 demonstrating that glucose was mainly consumed for ethanol production. At 10% solids loading (Figure 5b), the maximum ethanol concentration was 19.96 and 24.98 g/L corresponding to 50% and 62.5% yield in LP-3 and LP-4, respectively. Glucose increased rapidly during the first 24 hours and remained at a stable level for 24 hours after which it decreased. 8.32 and 14.09 g/L of glucose remained in LP-3 and LP-4 after 96 hours of fermentation. 0.48 g/L and 0.56 g/L of glycerol were formed respectively in LP-3 and LP-4.

### Comparative analysis of SSF with commercial enzymes

**Figure 6:**
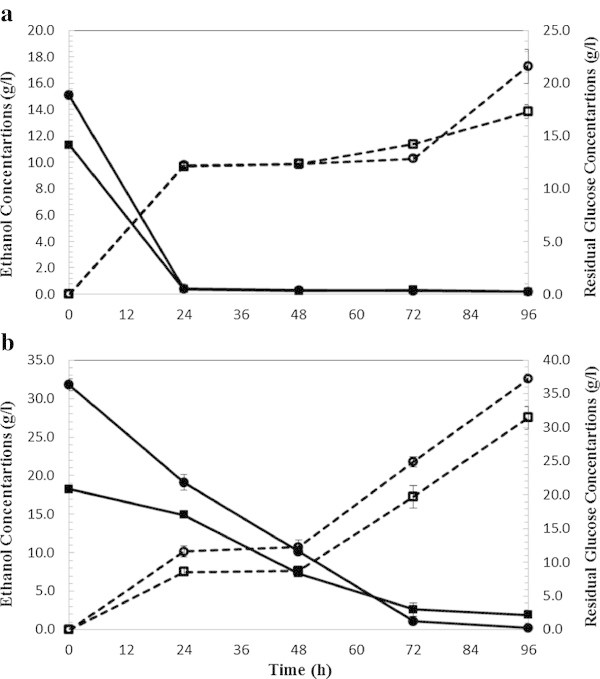
**SSF ethanol production (dashed lines) and residual glucose (bold lines) from wet exploded corn stover using commercial enzymes (a) at 5% solid loading and enzyme loading 5 FPU (squared label) and 15 FPU/g glucan (circled label) (b) at 10% solid loading and enzyme loading 5 FPU (squared label) and 15 FPU/g glucan (circled label).**

**Figure 7:**
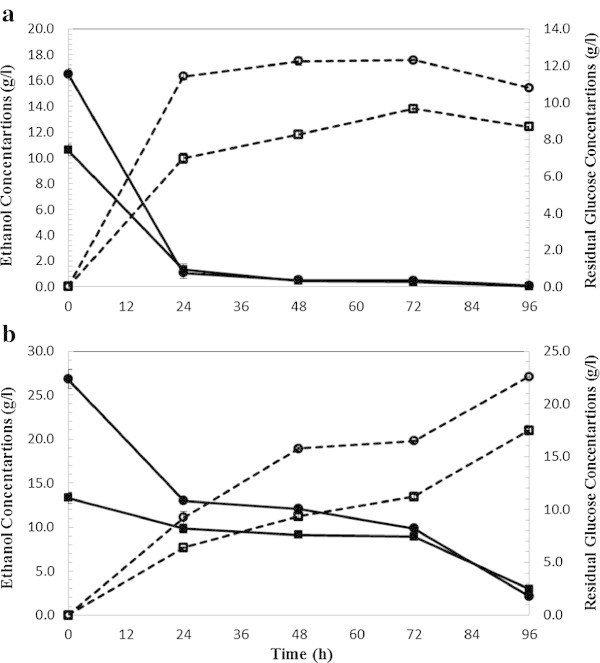
**SSF ethanol production (dashed lines) and residual glucose (bold lines) from wet exploded Loblolly pine using commercial enzymes. (a)** At 5% solid loading and enzyme loading 5 FPU (squared label) and 15 FPU/g glucan (circled label). **(b)** At 10% solid loading and enzyme loading 5 FPU (squared label) and 15 FPU/g glucan (circled label).

In order to compare ethanol production in the SSF process with commercial enzymes (celluclast 1.5 L and Novozym 188) and in-house produced enzymes (*T. reesei* and *A. saccharolyticus*), SSF runs were conducted on WECS and WELP (Figures 6 and 7) at 5 and 10% solids loadings. Several authors have agreed on the feasibility of SSF process at high solid loading (10% w/w) (Ballesteros et al. 2004; Linde et al. 2006; Öhgren et al. 2006). Fermentations of WECS and WELP hydrolyzed with commercial and on-site produced enzymes were compared under identical conditions.

The highest ethanol from wet exploded corn stover was achieved at 15 FPU/ g glucan and 5% solids loadings (75% and 84% of theoretical yield for in-house and commercial enzymes, respectively). The highest ethanol from wet exploded loblolly pine was achieved at 15 FPU/g glucan and 5% solids loadings (67% and 77% for in-house and commercial enzymes, respectively).

## Discussion

In this study, cellulolytic enzymes were produced in-house using *T. reesei* Rut C30 and *A. saccharolyticus* to reduce the cost of enzymatic hydrolysis and still keep high hydrolysis efficiency. Our results showed that we had all the necessary cellulolytic activities by combining the two selected fungal species. In-house produced enzymes showed higher filter paper activity (FPA), 4.8FPU/mL from *T. reesei* Rut C30 compared to other authors (Juhász et al. 2005; Kovács et al. 2009). In contrast to the huge difference in FPAs for *T. reesei* Rut C30 and commercial Celluclast 1.5 L (4.5 and 80 FPU/mL, respectively), the measured β-glucosidase activity of the *A. sacchar*olyticus was about half of Novozym 188. This is possibly due to the fact that β-glucosidase activity is measured on a simple substrate and not influenced by other enzyme activities, whereas FPA is determined on a complex substrate where several different bonds needs to be cleaved by different enzymes (Kovács et al. 2009).

In this study, in-house cellulase performance was compared with the traditional cellulase mixture, Celluclast 1.5 L and Novozym 188 instead of newer commercial cellulase preparations because recipe of new enzymes formulation is not known and also these enzymes’ performance is a result of multiple accessory enzymes present in the formulation (Rana et al. 2014). Therefore for the purpose of the study, comparison was made with known cellulase mixture, Celluclast and Novozym. The selection of a maximum of 10% solids loading was supported by previous findings that the better fermentation results are obtained when the solids loading is limited to 10% (Varga et al. 2004). In our preliminary studies with higher solids loading (20%) similar results were found and due to mixing problems and mass transfer issues only low levels of fermentation products were obtained (data not shown). Stenberg et al. (Stenberg et al. 2000) found similar limitations with steam-pretreated softwood. Other authors who were able to successfully apply a higher solids loading (>10%) used commercial enzymes with higher loadings (20–45 FPU/g glucan) (Linde et al. 2007; Stenberg et al. 2000; Varga et al. 2004).

In SHF, the maximally achieved theoretical ethanol yield were 70.2% and 62.5% from WECS and WELP, respectively, using 5% solids loading and 15 FPU/g glucan after 96 hours of fermentation. At the higher solids loading (10%) of WECS and WELP, sugar consumption was slow and delayed probably because of the presence of higher amounts of inhibitors in the hydrolyzate. Yeasts are not capable of metabolizing acids such as acetic acid under anaerobic conditions, but they are able to reduce the pentose and hexose driven aldehydic inhibitors (furfural and HMF) to their corresponding alcohols and this leads to reduced ethanol productivity until all the furfural/HMF has been consumed (Taherzadeh et al. 1999). Complete assimilation of these aldehydes takes time and results in a long lag phase before ethanol production (Liu et al. 2004; Taherzadeh et al. 2000).

In SSF, the highest achieved theoretical ethanol yields were 75.9% and 67.1% from WECS and WELP, respectively, using 5% solids loading and 15 FPU/g glucan after 96 hours of fermentation. Similar to SHF, at high solids loading, 10% (Figure 4b), the higher amounts of inhibitors were present in the medium which might have influenced the cells growth significantly as demonstrated by an increased lag phase (Taherzadeh et al. 2000). As can be seen in Figure 4b and 5b with solids loading 10%, the resulting ethanol concentration was higher than 5% SL but the yield was low considering the concentration of sugar. A high increase in glucose concentration can be observed at the beginning of SSF (until 48 hrs). Glucose concentration increased during the first 24 hours indicating that the hydrolysis rate superseded the fermentation rate. Stenberg et al. also observed the lag phase during SSF of softwood (Stenberg et al. 2000) at high solids loading with WIS concentrations of 10%. They found that 10% WIS fermentation did not start within 96 hrs. Yields at higher solids loading (10%) was slightly lower compared to 5% solid loading; however, the higher the solid loading during SSF, the lower the energy demand will be for the downstream processes such as distillation and concentration (Nguyen and Saddler 1991; Sun and Cheng 2002; Wingren et al. 2003). In other words, both high yield and higher ethanol concentration could be achieved by using higher solids loadings during SSF which will decrease the cost of production (von Sivers and Zacchi 1996; Wingren et al. 2003).

Generally our results are in the agreement with previous studies. Varga et al. (Varga et al. 2004) performed SSF with 12% solids loading and 22 FPU/g glucan resulting in an ethanol yield of 75%. Öhgren et al. (Öhgren et al. 2006) reported 73% ethanol yield from SSF of corn stover at 10% WIS and 25 FPU/g glucan using baker’s yeast. In contrary, using nearly similar WIS loading (9%), Sassner et al. (Sassner et al. 2006) found 20% ethanol yield from SSF acid impregnated and steam pretreated salix using 20 FPU/g glucan using baker’s yeast. One of the advantages of wet explosion pretreatment is formation of less inhibitors which makes it favorable for yeast fermentation with lower enzyme addition.

Compared to SHF, ethanol production in SSF was substantially faster demonstrating lower enzyme inhibition by glucose. Wet explosion pretreatment and in-house produced enzymes (from *T. reesei* and *A. saccharolyticus*) were the catalyst responsible for achieving the higher ethanol concentration. Moreover, the most efficient configuration will be realized when both hydrolysis and fermentation share common optimal conditions. So far, there is no reported study on comparison of SHF and SSF of wet exploded corn stover and loblolly pine. However, in one study on alkaline-wet oxidized corn stover by Varga et al. (Varga et al. 2004) an overall ethanol yield of 84% was achieved at 12% solids loading and 43.5 FPU/g glucan enzyme loading. In other study on SSF of SO_2_-impregnated and steam pretreated spruce by Sternberg et al. (Stenberg et al. 2000), highest ethanol yield, 82% (from glucose and mannose combined) was achieved at 5% solids loading and 32 FPU/g glucan. High enzyme loading is self-explanatory for higher yields of ethanol. Considering the cost of bioethanol production, cellulase cost is a critical parameter for process and cost improvement (Mielenz 2001). In this study we have minimized the addition of cellulases to demonstrate the possibilities of lowering the use of cellulase enzymes when using the wet explosion pretreatment for both corn stover and loblolly pine as feedstock.

Comparison between separate hydrolysis and fermentation (SHF) and simultaneous saccharification and fermentation (SSF) has resulted in the finding that SSF was more efficient compared to SHF, despite using a lower reaction temperature which is suboptimal for the enzyme hydrolysis reaction. The highest ethanol yields (75.98% (WECS) and 67.07% (WELP)) were achieved when WECS and WELP were hydrolyzed with 15 FPU/g glucan at 5% solids loading through a SSF process. Ethanol yield from loblolly pine was lower compared to corn stover in all fermentation experiments probably due to the presence of high concentrations of lignin in loblolly pine (the amount of lignin is nearly double compared to corn stover (Rana et al. 2012; Rana et al. 2013)). Lignin has been found to deactivate the action of enzymes by several researchers. Comparison of SSF with in-house enzymes and commercial enzymes showed slightly higher ethanol concentrations using commercial enzymes because of a faster cellulose conversion to glucose. This was probably due to additional accessory enzymes in the commercial product and also removal of undesired substances by purification in the commercial enzymes. Interestingly, we found no significant difference in the overall ethanol yields for samples hydrolyzed with commercial or on-site enzymes. These results indicate that there is a great potential for using in-house enzymes produced from for instance *T. reesei* and *A. saccharolyticus* as a substitute to using expensive commercial enzymes. Further work is needed to make this process more commercially attractive including purification and further concentration of in-house produced enzyme and using higher concentrations of pretreated solids and higher overall ethanol yields as well as optimization of some operational conditions.
